# Proton Conductive Channel Optimization in Methanol Resistive Hybrid Hyperbranched Polyamide Proton Exchange Membrane

**DOI:** 10.3390/polym9120703

**Published:** 2017-12-14

**Authors:** Liying Ma, Jing Li, Jie Xiong, Guoxiao Xu, Zhao Liu, Weiwei Cai

**Affiliations:** 1 School of Chemistry and Materials Science, Guizhou Normal University, 116 Baoshan North Road, Guiyang 550001, China; maliying2018@163.com; 2 Sustainable Energy Laboratory, Faculty of Materials Science and Chemistry, China University of Geosciences Wuhan, 388 Lumo RD, Wuhan 430074, China; cumtbxiongjie@163.com (J.X.); guoxiao_xu123@163.com (G.X.); 15629023972@163.com (Z.L.); 3 College of Chemistry, Chemical Engineering and Material Science, Zaozhuang University, Zaozhuang 277160, China

**Keywords:** proton exchange membrane, methanol resistivity, direct methanol fuel cells, hybrid membrane

## Abstract

Based on a previously developed polyamide proton conductive macromolecule, the nano-scale structure of the self-assembled proton conductive channels (PCCs) is adjusted via enlarging the nano-scale pore size within the macromolecules. Hyperbranched polyamide macromolecules with different size are synthesized from different monomers to tune the nano-scale pore size within the macromolecules, and a series of hybrid membranes are prepared from these two micromoles to optimize the PCC structure in the proton exchange membrane. The optimized membrane exhibits methanol permeability low to 2.2 × 10^−7^ cm^2^/s, while the proton conductivity of the hybrid membrane can reach 0.25 S/cm at 80 °C, which was much higher than the value of the Nafion 117 membrane (0.192 S/cm). By considering the mechanical, dimensional, and the thermal properties, the hybrid hyperbranched polyamide proton exchange membrane (PEM) exhibits promising application potential in direct methanol fuel cells (DMFC).

## 1. Introduction

Direct methanol fuel cells (DMFCs) are regarded as promising power sources for stationary, automotive, and portable electronic applications [1,2,3,4,5,6] attributed to the advantages, including facile fuel storage and utilization, mild operating temperature, and high energy density. Proton exchange membrane (PEM), which not only separates the fuel and oxidant, but also conducts protons from anode to cathode, is one of the most key components in a DMFC [7]. The perfluorinated sulfonic acidic Nafion membrane (DuPont) is the currently commercial proton exchange membrane for fuel cell applications, owing to the high proton conductivity under relatively low temperature, satisfied mechanical strength, and excellent oxidative stabilities. However, the high price and the high methanol permeability of the Nafion membrane are the major limitations that hinder the large scale commercial applications of DMFC technology [8,9,10,11,12]. It was reported that more than 40% of the methanol fuel was wasted through leaking crossover the Nafion membrane during the DMFC operation due to that the Nafion molecules with long side chains cannot be densely assembled [13,14]. The permeated methanol would be oxidized in the cathode catalyst layer, and hence, lower the power density of DMFC [15]. Therefore, many studies on alternative PEM material development were conducted to overcome the methanol permeation issue [16,17,18,19]. By considering the material cost simultaneously, great efforts have be made to develop high performance sulfonated aromatic polymers, such as poly(ether ether ketone) [20,21,22], poly(ether ketone) (PEK) [23,24], polybenzimidazole [25,26], and polyimide [27,28]. Zhang et al. [22] prepared a serial of sulfonated poly(ether ether ketone)s with pendant benzimidazole groups, the membranes exhibited satisfied mechanical and thermal stabilities, while the proton conductivity of the membranes was not ideal. Seo et al. [24] synthesized several PEKs and showed higher water uptake, IECs and thermal stability, while the proton conductivity of the membranes was still lower than that of Nafion 211.

Well-connected and highly ordered proton conductive channel (PCC) is considered to be the most key factor for high proton conductivity, strong mechanical property and low fuel permeability at the same time. To establish high efficient PCCs by controlling the alignment in the PEMs is therefore one of the most important tasks for novel PEM development [29]. Okamoto et al. [30] constructed well-connected PCCs in polyimide block copolymers, which was originated from hydrophobic and hydrophilic micro-phase separation by tuning the position of sulfonic acid groups on the polymer backbone or side-chain. These PEMs showed two times higher proton conductivity (25 mS/cm at 30 °C and 50% RH) than the random analogs. Li et al. [31] synthesized triblock copolymers with in-situ formed well-connected PCCs for efficient proton conduction due to the nano-phase separation between the hydrophilic groups and the hydrophobic chain. The membrane exhibited high proton conductivity under reduced relative humidity conditions (RH = 30–50%), superior antioxidative, and thermal stabilities. Another example is that the graft copolymers can possess higher proton conductivity and stronger mechanical strength than block copolymer due to the different degree of anisotropic PCCs in PEMs [32]. Guiver et al. [33] synthesized a comb-shaped graft polymer with densely sulfonated side-chains, and the densely sulfonated chains formed self-assemble nanoscale PCCs, resulting in a high proton conductivity (100 mS/cm at 90% RH). Therefore, constructing the PCCs and controlling its orientation in PEMs to provide the effective transport route is an interesting and worth investigating area [34].

In our previous studies, we developed a hyperbranched PEMs with self-assembled hierarchical PCC (HPCC) for DMFC application [35,36]. The PEMs exhibited remarkably improved methanol-permeation resistant property and proton conductive property. The HPCC in the membrane is consisted of the first-order PCCs (FOPCCs) formed by the dense –SO_3_H within the –COOH end-capped macromolecules, and the second-order PCCs (SOPCCs) that were formed by the intermolecular hydrogen bonds among the –COOH groups.

**Figure 1:**
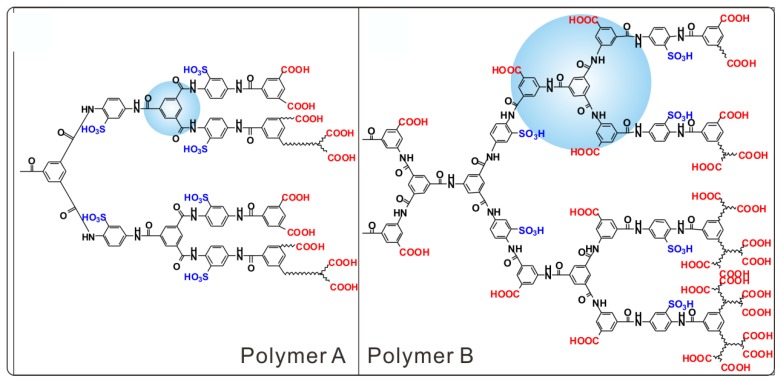
Chemical structure of polymer A and polymer B for first-order PCCs (FOPCC) optimization.

Here, in this work, nano-structure of the FOPCCs in the PEM membrane are adjust to optimize the hyperbranched PEM performance via enlarging the nano-scale pore size within the macromolecules to improve the proton conductive efficiency. Polymer B in Figure 1 was therefore designed to replace polymer A. Since the membrane made from polymer B is fragile due to the rigidity from multiple benzene ring structure in the membrane, hybrid membranes with different weight ratio of polymer A to polymer B are fabricated. The optimized membrane exhibits a methanol permeability of low to 2.2 × 10^−7^ cm^2^/s. At the same time, the proton conductivity of the hybrid membrane of this work is as high as 0.25 S/cm at 80 °C, much higher than the value of the Nafion 117 membrane (0.192 S/cm) [36]. By considering the mechanical, dimensional, and the thermal properties, the hybrid hyperbranched polyamide PEM exhibits promising application potential in DMFC.

## 2. Experimental

### 2.1. Materials

Trimesic acid (TA), 2,4-diaminobenzenesulfonic acid (DSA), and Dimethyl 5-aminoisophthalate (DAP) were purchased from Adamas Reagent, Ltd., and dried at 80 °C for 24 h before using. *N*-Methyl-2-pyrrolidone (NMP), triphenylphosphosphite (TPP), and Pyridine (Py) were provided by Sinopharm Chemical Reagent Co., Ltd. and distilled from KOH. Nafion 117 was supplied by Dupont Co. (Wilmington, DE, USA).

### 2.2. Synthesis

#### 2.2.1. Polymer A

**Scheme 1:**
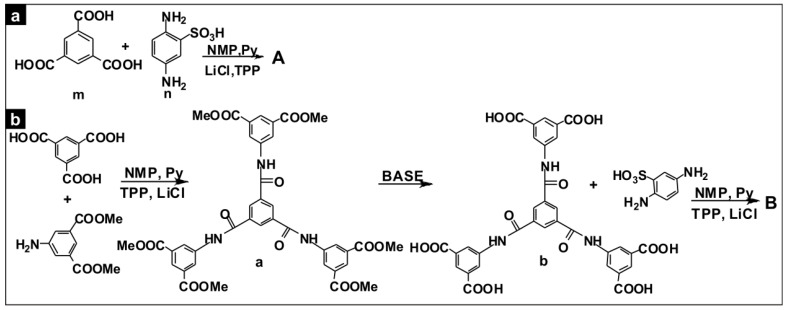
Synthesis route of polymer A (**a**) and B (**b**).

TPP (1 mL), Py (3 mL), and NMP (4 mL) were added into a 50 mL two-necked round-bottom flask with TA (2 mmol), LiCl (0.32 g) and p-DSA (2 mmol). The mixture was maintained 100 °C for 2 h under argon atmosphere. Upon completion, the mixture was cooled to room temperature, and then washed with methanol and ultrapure water for several times. The product was dried at 80 °C under vacuum for 24 h (Scheme 1).

#### 2.2.2. Polymer B

Synthesis of carboxylic ester (*a*): TA (10 mmol), DAP (20 mmol), TPP (5 mL), NMP (20 mL), and Py (20 mL) were added into a 100 mL two-necked round-bottom flask, and the reaction was maintained at 100 °C overnight. After cooling to RT, the resulting solution was poured into 100 mL methanol, filtered and washed with methanol and water, and then dried at 80 °C for 24 h (Scheme 1).

Synthesis of carboxylic acid (*b*): carboxylic acid was synthesized by a basic hydrolysis reaction of product *a* (5 mmol), KOH (30 mmol), and THF (40 mL) at room temperature for 1 d. The clear liquid was acidized with hydrochloric acid till the pH = 3, and then the THF and water were removed from the reaction solution. The white powder was dried under vacuum at 80 °C for 24 h (Scheme 1).

Synthesis of Polymer B: Product *b* (2 mmol), p-DSA (2 mmol), LiCl (0.32 g), TPP (1 mL), NMP (4 mL), and Py (3 mL) were added into a flask that was equipped with an argon inlet, magnetic stirrer, and water-cooled condenser. The reaction mixture was maintained at 100 °C for 3 h. The viscous polymer was washed with methanol and water several times and dried at 80 °C under vacuum for 24 h (Scheme 1).

For both Polymer A and Polymer B, the size of the hyperbranded macromolecules can be determined by precisely control the monomer ratio. At the same time, the successful –COOH end-capping of these two polymers can be achieved since the monomer with –COOH groups are slightly excessive.

### 2.3. Membrane Preparation

The hybrid membranes of ca. 100 um were prepared using solution casting technique. The polymer A and the polymer B were dissolved in dimethyl sulfoxide (DMSO) to form homogeneous solution at about 80 °C, respectively. Then, the hybrid solution of polymer A and polymer B were filtered and cast on a Petri dish and evaporated the solvent at 80 °C to give a uniform hybrid membrane. AB-*x* membranes with *x* as the weight percentage of the polymer A in the hybrid membranes (*x* = 80, 85, 90, 95). The membranes were immersed in 1 M HCl solution for acid form by ion exchange at RT for 12 h, and then washed with water until the pH of the solutions reached pH = 7.

### 2.4. Measurements and Characterizations

^1^H NMR spectra were carried out using a Bruker AVANCE III HD instrument (400 MHz) in DMSO-*d*_6_ as solvent and TMS as the internal standard. The polymers structure was recorded on a FT-IR Nicolet6700 spectrometer. The atomic force microscopy (AFM) images of the hybrid membranes were carried out using a Digital Instruments Multimode (SPM9700, Shimadzu, Kyoto, Japan). The cross-section images of the hybrid membranes were obtained from a scanning electron microscopy (SEM, SU8010, Hitachi, Tokyo, Japan). The thermogravimetric analysis (TGA) of membranes was performed with a NetzschSTA 409 PC TG-DTA instrument (Netzsch, Bavaria, Germany) in the temperature range from 30 °C to 600 °C, at 10 °C/min under a nitrogen atmosphere. Proton conductivity of the hybrid membranes was performed by four-electrode AC impendence method using an Interface 1000 electrochemical workstation (Gamry, Warminster, PA, USA). The membrane (1 cm × 3 cm) was placed in the test cell and measured in the temperature range of 25–80 °C at 100% RH. The proton conductivity was calculated from the following equation:

(1) $$\sigma =\frac{d}{Rtw}$$

where *σ* is the proton conductivity in the transverse direction of the membranes (S/cm), *w* and *t* are the membrane width (cm) and thickness (cm), respectively. *d* is the distance between the two electrodes (cm). *R* is the measured resistance of the membrane (Ω).

Ion exchange capacity (IEC) of the membranes was determined by a classical acid-base titration. Membrane sample was immersed in 1 M NaCl solution for 48 h to replace the H^+^ ions with Na^+^ ions. The exchange proton H^+^ in the solution was titrated using 0.01 M NaOH, with phenolphthalein as an indicator. The IEC was calculated by the equation as follows:

(2) $$\mathrm{IEC}=\frac{V\times C}{W}\left(\mathrm{mmol}/g\right)$$

where C is the concentration of the 0.01 M NaOH solution (mol/L), V and W is the volume of the 0.01 M NaOH solution (mL) and the weight of dry membrane (g), respectively.

A piece of membrane sample was immersed in deionized water at room temperature for 12 h, and then removed the surface water with tissue paper and recorded the weight and the volume quickly. The membrane was then dried at 80 °C for 12 h to remove the residual solvent or water and quickly recorded the weight and the volume. Water uptake and volume swelling of the membranes was calculated by the following equation:

(3) $$\mathrm{WU}=\frac{\left(W_{Wet}-W_{dry}\right)}{W_{dry}}\times 100\%$$

where W_dry_ and W_wet_ are the weights of dry and wet membranes, respectively.

(4) $$VS=\frac{V_{Wet}-V_{dry}}{V_{dry}}\times 100\%$$

where V_dry_ and V_wet_ are the volumes of the corresponding dry and wet membranes, respectively.

The methanol permeability of membranes was measured by using an “H” type diaphragm diffusion with two cells separated by the membrane. One cell contained 1 M methanol solution and the other contained deionized water with magnetic stirrer for methanol diffusion. The methanol concentration in the water side of the diffusion cell was measured by using a gas chromatograph (9790II Zhejiang Fuli Analytical Instruments Co., Ltd., Wenling, China). The methanol permeability was determined according to the following equation:

(5) $$p=\frac{l}{A}\times \frac{V_{B}}{C_{A}}\times \frac{\Delta C}{\Delta t}$$

where *p* is the methanol permeability (cm^2^/s), l and A is the thickness and the effective diffusion area, respectively. V_B_ is the volume of the compartment (cm^3^), C_A_ is the initial concentration of methanol (mol/cm^3^). ΔC and Δt are methanol concentration of the methanol solution (mol/cm^3^) and the diffusion time (s), respectively.

Strain-tensile cuves of the hybrid membranes and Nafion membrane (water soaked) were recorded using Labthink XLW (PC) at a strain rate of 25 mm/min with a 50 N load cell.

## 3. Results and Discussion

**Figure 2:**
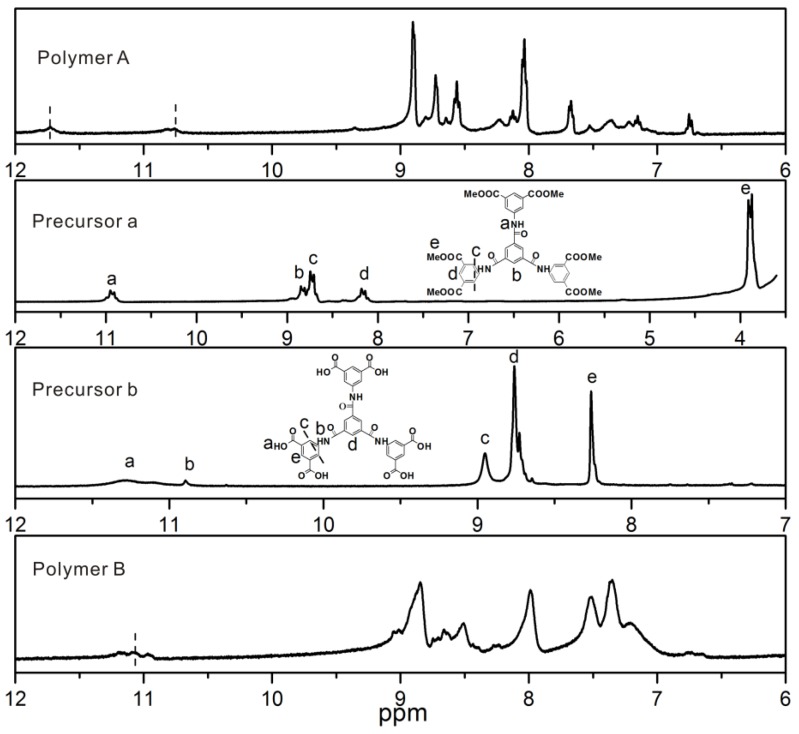
^1^H NMR spectra of precursor *a* and *b*, Polymer A and polymer B.

**Figure 3:**
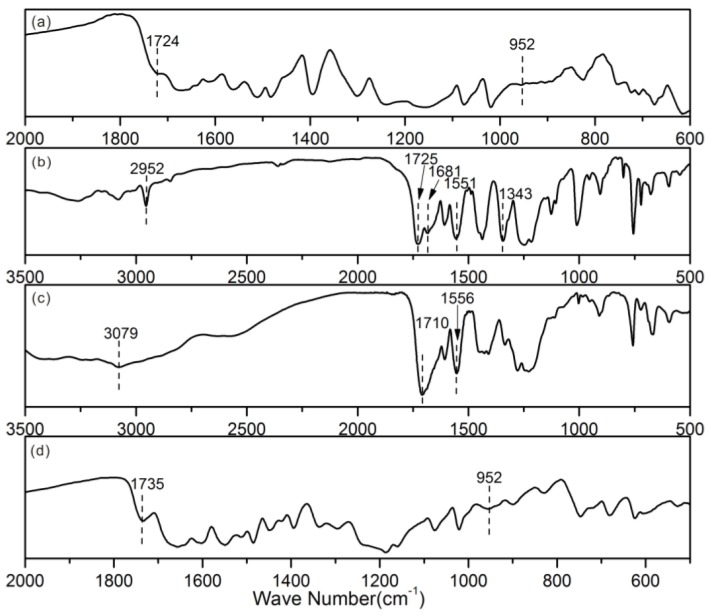
FT-IR spectra of (**a**) Polymer A; (**b**) precursor *a*; (**c**) precursor *b*; and (**d**) Polymer B.

Polymer structure of polymer A and polymer B was confirmed by ^1^H NMR and FT-IR spectra in Figure 2 and Figure 3, respectively. For polymer A, ^1^H NMR (400 MHz, DMSO-d_6_), the distinct resonance at 11.8 ppm and 10.9 ppm presented the –COOH and the –CONH–group, respectively. For the precursor a, the signals of hydrogen (Ha) are assigned to the –CONH–, the signals of hydrogen atoms (H_b–d_) are attributed to the phenyl group, and the signals of hydrogen (H_e_) are assigned to methyl group. For the precursor b, ^1^H NMR (400 MHz, DMSO-d_6_) δ 11.3, 10.9, 8.9, 8.8, 8.3 ascribing to the hydrogen atom on the molecule marked by “a”, “b”, “c”, “d”, and “e”, respectively. For polymer B, the signals at 11.1 ppm presented the end-capped group –COOH of polymer.

For polymer A (Figure 3a), the FT-IR spectrum depicted the C=O stretching bands at about 1700 cm^−1^ and O–H bending vibrations at 945 cm^−1^ in –COOH of polymer A. For precursor *a* (Figure 3b), the peak at 2952 cm^−1^ are assigned to the amide group, the 1725 and 1681 cm^−1^ indicate the presence of the C=O of carboxyl and amide, respectively. The peak at 1551 cm^−1^ is attributed to the N–H bending vibrations, and 1343 cm^−1^ are assigned to the C–N stretching vibrations. For precursor b (Figure 3c), 3079 cm^−1^ (υ_OH_, –COOH), 1710 cm^−1^ (υ_C=O_, –CONH), and 1556 cm^−1^ (υ_C=O_, –COOH). For polymer B (Figure 3d), the band at 1735 cm^−1^ and 952 cm^−1^ indicated the presence of the C=O stretching vibration and O–H bending vibration, respectively.

**Figure 4:**
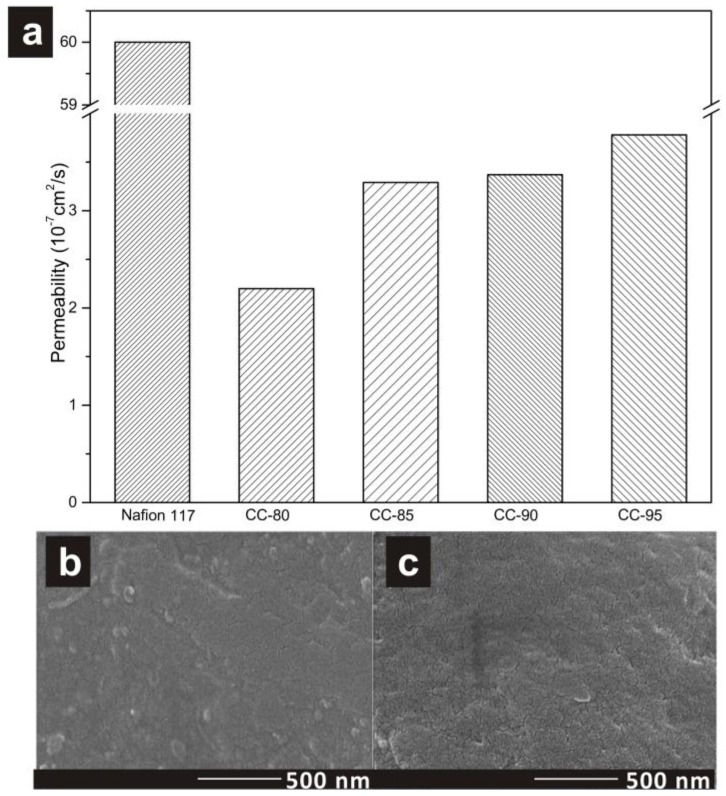
The methanol permeability through the hybrid membranes (**a**) and the cross-section SEM images of the CC-80 (**b**) and CC-95 (**c**) hybrid membranes.

Methanol permeability is the key descriptor of a PEM for DMFC application. Methanol permeability through the hybrid membranes are compared in Figure 4a. The hybrid membrane exhibited methanol permeability in the range of 2.20 × 10^−7^ cm^2^/s to 3.78 × 10^−7^ cm^2^/s, more than 1.5 times lower than that of CC-100 membrane made from polymer A. Apparently, with the polymer B weight ratio ranging from 0 to 20%, the methanol permeability diminished from 4.86 × 10^−7^ to 2.20 × 10^−7^ cm^2^/s, attributed to the well-connected network resulting from hydrogen bonding of abundant –SO_3_H group and end-capped –COOH group in polymer A and B. The well-connected network effectively prevented methanol permeation, Figure 4b,c illustrated the scanning electron microscope morphologies (SEM) of the cross-section of CC-80 and CC-95 hybrid membranes, which showed a flat and uniform topography resulting in effective resistance on methanol permeation.

**Figure 5:**
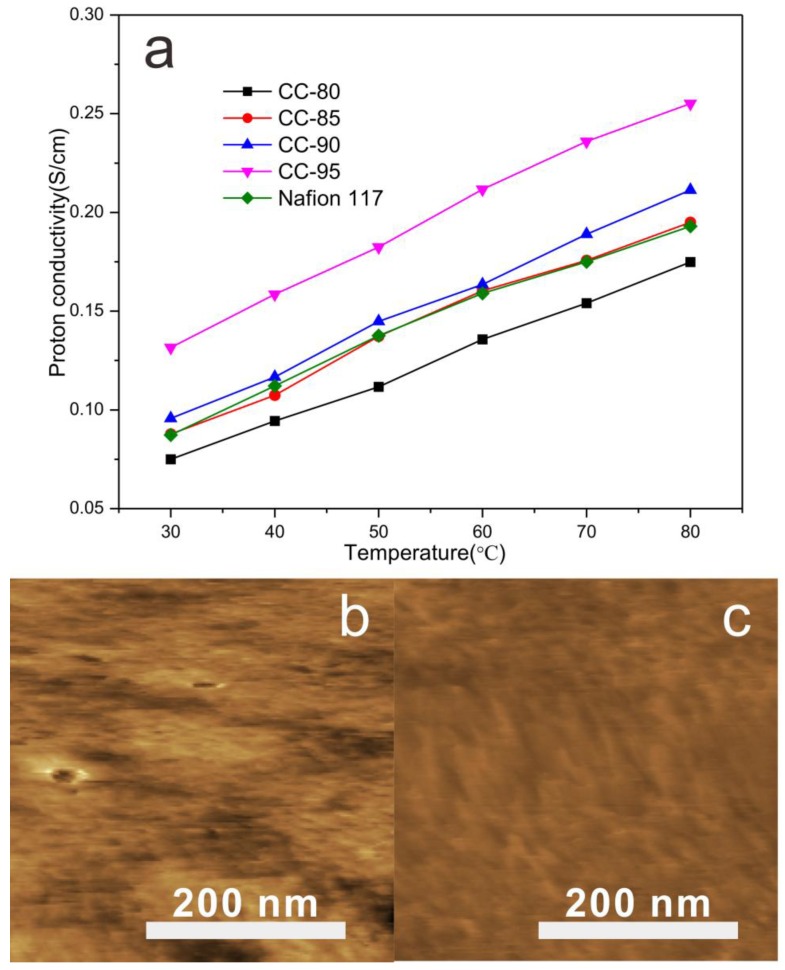
(**a**) Temperature dependences of proton conductivity of the hybrid membranes and Nafion 117; the AFM tapping images of the (**b**) CC-80 and (**c**) CC-95 hybrid membranes.

Proton conductivity is considered as the most important coefficient to evaluate a PEM. Displayed in Figure 5 are temperature dependency curves of proton conductivity for the four hybrid membranes and the Nafion 117 membrane. It can be found that the CC-90 and CC-95 hybrid membranes exhibited superior proton conductive property than the Nafion 117 membrane in the entire studied temperature range. The CC-85 displayed comparative proton conductivity with that of Nafion 117 membrane. The proton conductivity of the CC-95 membrane is high to 0.132 S/cm and 0.255 S/cm at 30 °C and 80 °C, respectively. The great proton conductivity of the CC-95 membrane agrees well with the high measured IEC value, as provided in Table 1. With the increasing ratio of polymer A from 80% to 95%, the IEC value of the hybrid membranes increased from 1.52 to 1.93 mmol/g. At the same, proton conductivity of the hybrid membrane may also relate to the micro phase-separation structure of the membranes. As compared in Figure 5b,c, the AFM tapping mode phase image of the CC-95 hybrid membrane with the best proton conductive performance exhibited more remarkable hydrophilic/hydrophobic phase-separation than the CC-80 hybrid membrane did.

By considering proton conductive and methanol-permeation resistive properties at the same time, the selectivity of the hybrid membranes are calculated according to the equation *s* = *σ*/*p*, where *σ* and *p* are proton conductivity and methanol permeability of the membrane, respectively. It can be found from Table 1 That all the four hybrid membranes showed higher selectivity than pristine CC-100 membrane, primarily due to the depressed methanol permeation. Among the four hybrid membranes, the CC-95 membrane exhibited the greatest selectivity, and is therefore considered as the optimized CC-x hybrid membrane for DMFC application. Selectivity of the CC-95 membranes is 34.92 × 10^4^ S·s/cm^3^, ca. 1.5 times higher than that of the CC-100 membrane.

Before the practical application in DMFC, dimensional, mechanical, and thermal stabilities of the hybrid membranes have to be evaluated. The water uptake and volume swelling of the hybrid membranes were therefore measured and compared in Table 1. It can be found that the volume swelling of the hybrid membranes diminished with an increasing amount of polymer A due to the decreased water uptake ratio. Whatever, all of the hybrid membranes exhibited a smaller volume swelling ratio value than the water uptake value, indicating great dimensional stability of the hybrid membranes.

**Figure 6:**
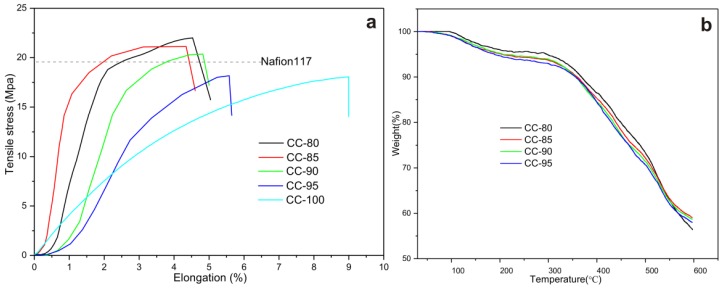
The stress-strain relationship (**a**) and the thermogravimetric analysis (TGA) curves (**b**) of the hybrid membranes.

Strong mechanical property of the membrane is one of the important demands to withstand high compression for MEA fabrication of PEMFC and DMFC applications [37]. The hybrid membranes exhibited good mechanical property from the stress-strain plots, as shown as displayed in Figure 6a. The hybrid membranes exhibited high tensile stress of 18–22 MPa, which can be comparable to that of Nafion 117 (20 MPa). Enhanced tensile stress of the hybrid membranes is attributed to from the hydrogen bonding interaction between the end-capped –COOH groups of macromolecules and the multi-benzene ring structure of polymer B. Good thermal stability of the hybrid membranes was also proved by the TGA analysis (Figure 6b). The hybrid membranes exhibited three-step weight loss thermograms in the TGA analysis. The first weight loss of the hybrid membranes at about 100 °C is attributed to the evaporation of absorbed water and solvent. The second between 250 °C and 300 °C was caused by the degradation of the sulfonic acid groups within the membranes. The third was seen at above 550 °C due to the degradation of the polymer main chain [38]. In conclusion, all the three prepared exhibited satisfied mechanical and thermal stabilities for fuel cell applications.

## 4. Conclusions

Two –COOH end-capped hyperbranched polymers were successfully synthesized and hybrid proton exchange membranes were prepared from them with different ratios. The obtained hybrid membrane exhibited great proton conductivity, strong mechanical property, low methanol permeability, and satisfied thermal stability for DMFC application. The optimized hybrid membrane exhibited a methanol permeability of 3.78 × 10^−7^ cm^2^/s, which is ca. 15 times lower than that of the Nafion 117 membrane. At the same time, the proton conductivity of this optimized membrane was 0.255 S/cm at 80 °C, which is also much greater than that of the commercial Nafion 117 membrane (0.193 S/cm at 80 °C). Above all, with satisfied thermal and mechanical stabilities, the prepared hybrid membrane showed promising potential for application in DMFCs.

## Figures and Tables

**Table 1:** Ion exchange capacity (IEC), water uptake, volume swelling, methanol permeability and selectivity of hybrid membranes.

Membranes	CC-80	CC-85	CC-90	CC-95	CC-100
Water uptake (%)	37.56	36.27	35.25	33.52	31.71
Volume swelling (%)	34.95	32.02	31.19	30.41	27.48
Permeability (10^−7^ cm^2^/s)	2.20	3.29	3.37	3.78	4.86
IEC (mmol/g)	1.5243	1.7293	1.8905	1.9263	2.33
Proton conductivity (S/cm, 30 °C)	0.075	0.088	0.096	0.132	0.113
Selectivity (10^4^ S·s/cm^3^)	34.09	26.75	28.49	34.92	23.25
